# Tremors in cats with hepatic encephalopathy‐congenital portosystemic shunts or postattenuation neurological syndrome

**DOI:** 10.1002/vetr.4746

**Published:** 2024-11-14

**Authors:** Theofanis Liatis, Sofie F. M. Bhatti, Barbara Glanemann, Steven De Decker

**Affiliations:** ^1^ Department of Clinical Science and Services Royal Veterinary College Hatfield UK; ^2^ Small Animal Department, Small Animal Teaching Hospital, Faculty of Veterinary Medicine Ghent University Merelbeke Belgium

**Keywords:** ammonia, asterixis, Holmes tremor, manganese, parkinsonian‐like syndrome

## Abstract

**Background:**

Tremors have been reported as a neurological sign in cats with hepatic encephalopathy due to congenital portosystemic shunts (HE‐CPSS) or postattenuation neurological syndrome (PANS).

**Methods:**

The clinical records of cats diagnosed with HE‐CPSS and manifesting tremors between 2003 and 2023 were retrospectively reviewed to characterise the clinical features of the tremors.

**Results:**

Nineteen cats with HE‐CPSS were included, of which 17 manifested tremors at admission and two had PANS. Domestic shorthair was the most common breed (12/19). Tremors were the only neurological sign in six of the 19 cats. Tremor localisation was generalised (10/19) or focal to the head (8/19) or a limb (1/19). Most tremors were episodic and non‐intentional (15/19), occurring usually at rest with occasional intentional features (4/19). Of the 14 cats for which 1‐month follow‐up was available, tremors discontinued in nine after surgical or medical management.

**Limitations:**

The limitations of this study included its retrospective nature and the lack of video recordings, brain magnetic resonance imaging and electrophysiological evaluation of tremors in all cases.

**Conclusions:**

A diagnosis of HE‐CPSS or PANS should be considered in cats with generalised or focal tremors.

## INTRODUCTION

Tremor has been reported as an infrequent neurological sign in dogs and cats with hepatic encephalopathy due to congenital portosystemic shunts (HE‐CPSS)^1^, ^2^, ^3^, ^4^, ^5^ and in postattenuation neurological syndrome (PANS), which is a severe complication after surgical attenuation of congenital portosystemic shunts.^6^, ^7^ Hyperammonaemia and hypermanganesaemia are important clinicopathological changes in HE‐CPSS in dogs and cats.^8^, ^9^ Hyperammonaemia can cause astrocyte swelling contributing to HE,^9^ and when marked it might be associated with generalised tremors in dogs.^10^ Manganese is a neurotoxin that is considered to act synergistically with ammonia in HE^9^ and accumulates preferentially in the basal nuclei in people and dogs with HE.^11^, ^12^ In vivo, ammonia and manganese can have a synergistic negative effect on astrocytes by promoting free radical production, inner mitochondrial membrane depolarisation and loss of cellular integrity, whereas manganese alone can increase astrocyte expression of peripheral‐type benzodiazepine receptors, leading to synthesis of neurosteroids and an increased GABAergic tone.^9^

Although tremors have sporadically been reported in cats with HE‐CPSS,^1^, ^2^, ^4^ they have not yet been characterised in greater detail. The aim of this study was to describe the clinical features of tremors in cats with HE‐CPSS.

## MATERIALS AND METHODS

This was an observational single‐centre retrospective study conducted at a referral hospital between 1 January 2003 and 1 January 2023. Ethical approval was not required for this study due to its retrospective nature. Cases were identified by searching the hospital's electronic record database. Search terms included tremor, feline or cat, and hepatic encephalopathy or portosystemic shunt. To be eligible for inclusion in the study, cats needed to have complete medical records, a clinical diagnosis of HE‐CPSS (based on published criteria)^4^ and a tremor^13^ on neurological examination at admission or after surgery. Information extracted from the medical records of eligible cats included signalment, presenting complaints, neurological findings, serum ammonia concentration (if available) and final diagnosis. If available, brain magnetic resonance imaging (MRI), cerebrospinal fluid (CSF) analysis findings, video footage of tremors and follow‐up data were also collected. The onset of clinical signs was categorised into hyperacute (<24 hours), acute (1‒7 days), subacute (7‒15 days) and chronic (>15 days).^14^ All clinical and neurological examinations were performed by a board‐certified neurologist or a neurology resident under the direct supervision of a board‐certified neurologist. MRI used a high‐field magnet (1.5 T Intera, Philips Healthcare) and included a minimum of transverse and sagittal plane T2‐weighted, transverse fluid attenuation inversion recovery and T1‐weighted pre‐ and postcontrast (gadopentetate dimeglumine 0.1 mmol/kg IV bolus) images. Follow‐up was obtained from available clinical records (physical re‐examination or client communication). Descriptive statistical analysis was performed using standard statistical software (SPSS Statistics 26, IBM Corporation).

## RESULTS

One hundred and twenty‐two cats were found with confirmed portosystemic shunts. Nineteen cats (15.6%) met the inclusion criteria, of which 12 (63.2%)were domestic shorthair and seven (36.8%) were purebred. Of the purebred cats, three were British shorthair and the remaining four were Bengal, exotic shorthair, Maine coon or Persian. Twelve (63.2%) were male and seven (36.8%) were female; 12 cats (63.2%) were neutered and seven (36.8%) were entire. Median age at presentation was 7 months (range: 3 months‒9.6 years; interquartile range [IQR]: 1.1 years). Median bodyweight at presentation was 2.1 kg (range: 0.9‒3.5 kg; IQR: 1.5 kg).

The onset of clinical signs was either chronic (16/19; 84.2%), hyperacute (2/19; 10.5%) or acute (1/19; 5.3%). Progression of signs was seen in 17 of the 19 cats (89.5%), with two (10.5%) being static. Neurological signs were symmetrical in 18 (94.7%) cats and lateralised in one (5.3%) cat.

Main presenting complaints included hypersalivation (12/19; 63.2%), ataxia (11/19; 57.9%) and lethargy (9/19; 47.4%). Neuroanatomical localisation was either vestibulocerebellar (6/19; 31.6%), multifocal (5/19; 26.3%) or cerebellar (2/19; 10.5%). However, six (31.6%) cats had tremors as the only abnormality on neurological examination, with neuroanatomical localisation characterised as diffuse central nervous system. Tremors were present in 17 cats at admission (Video 1), while two cats developed tremors immediately after surgical attenuation. Tremor localisation was generalised (10/19; 52.6%) or focal localised to the head (8/19; 42.1%) or a single thoracic limb (1/19; 5.3%). Most tremors were episodic and non‐intentional (15/19; 78.9%), occurring usually at rest, with intentional features in some cases (4/19; 21%) (Supporting Information S1).

**VIDEO 1:** Tremors in three cats with hepatic encephalopathy due to congenital portosystemic shunts

**FIGURE 1:**
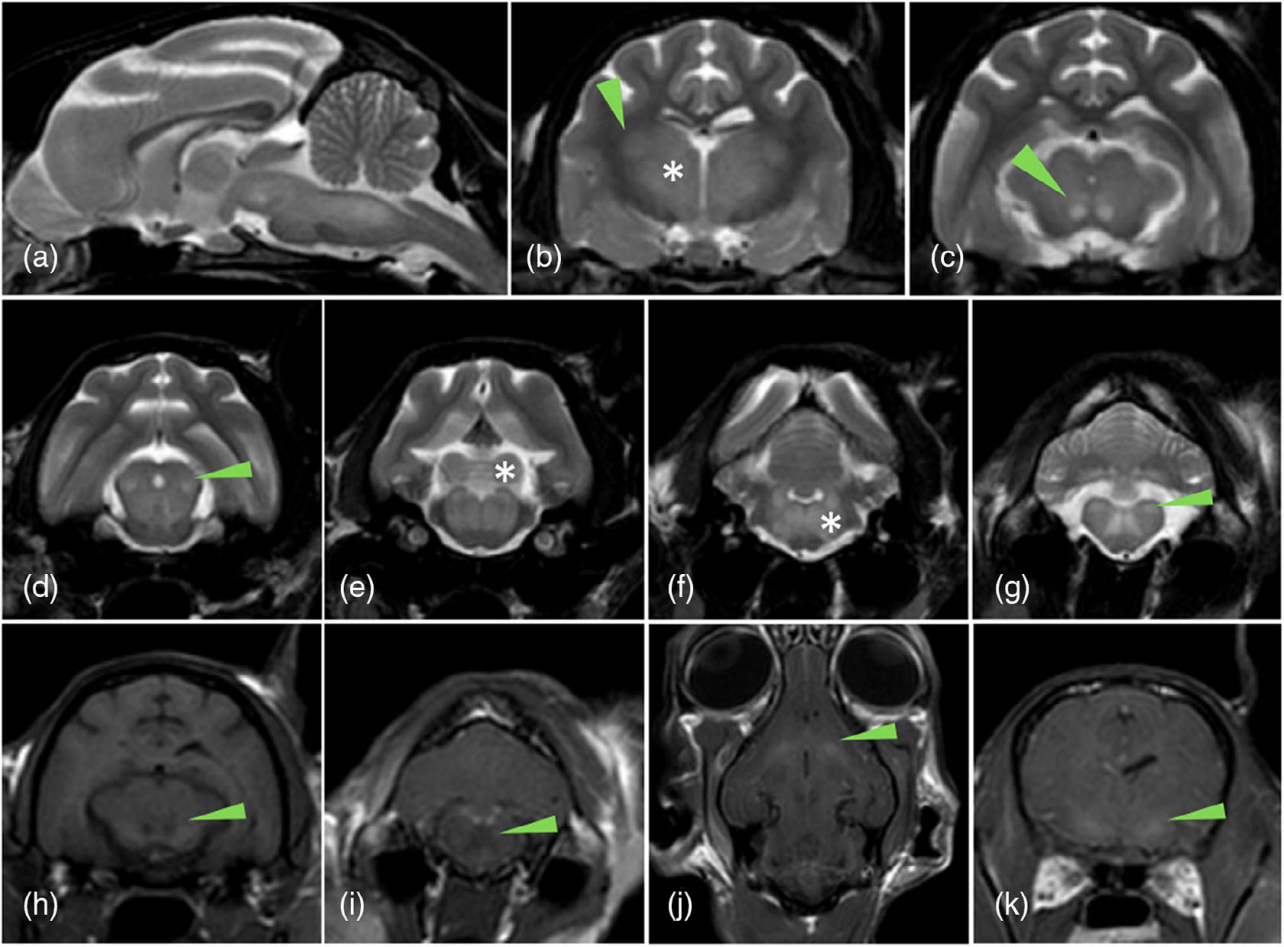
Magnetic resonance imaging of a cat with hepatic encephalopathy due to congenital extrahepatic portosystemic shunts revealed bilateral symmetrical T2‐weighted (T2W) hyperintense and T1‐weighted (T1W) hypointense non‐contrast‐enhancing lesions in the thalamus, lateral geniculate nuclei, red nuclei, caudal colliculi, vestibular nuclei, olivary nuclei and dentate nuclei, and T1W hyperintensity in the lentiform nuclei. (a) Sagittal T2W sequence showing diffuse T2W hyperintensity in the brainstem. Transverse T2W sequences showing bilateral symmetric T2W hyperintensities in the (b) thalami (asterisk) and lateral geniculate nuclei (arrowhead), (c) red nuclei (arrowhead), (d) rostral colliculi (arrowhead), (e) caudal colliculi (asterisk) and reticular formation, (f) white matter (asterisk) and (g) vestibular nuclei (arrowhead). Transverse (h and i), dorsal (j) and transverse (k) T1W precontrast sequences showing T1W bilateral hypointensity of the red nuclei (arrowhead; h), white matter (arrowhead; i) and T1W hyperintensity of the lentiform nuclei (arrowhead; k and l)

Hyperammonaemia was present in 12 of 15 (80%) cats that had ammonia tested. Confirmation of HE‐CPSS was based on multiple tests (Supporting Information S2). MRI of the brain was performed in two of 19 (10.5%) cats, of which one was normal and one abnormal (Figure 1). In that case, thiamine deficiency encephalopathy was not likely as the cat was on a balanced commercial diet. Cisternal cerebellomedullary CSF analysis was normal in the two cases in which it was performed. Electrodiagnostics were not performed. All cats but one were confirmed to have single (17/18; 94.4%) or multiple (1/18; 5.6%) extrahepatic portosystemic shunts, and two of them had PANS. The remaining cat had an intrahepatic portosystemic shunt. All cats received medical treatment, including lactulose and clinical diet (19/19; 100%), phenobarbital (9/19; 47.3%), amoxicillin‒clavulanate (9/19; 47.4%), levetiracetam (5/19; 26.3%) and ampicillin (3/19; 15.8%). Four cats (21%) were treated only medically. Fifteen cats were treated surgically with either partial (thin film band) ligation (12/15; 80%) or complete (prolene) ligation (3/15; 20%).

One‐month follow‐up information was available for 14 of the 19 cats (73.7%); 10 of 15 surgically treated cats and all cats with only medical treatment. Of the cats that were treated surgically, two were euthanased shortly after surgery due to deterioration, and three were lost to follow‐up (1 of which had PANS). In surgically treated cats that manifested tremors on admission (9/10), six of nine had their tremors resolved (five with partial and one with complete ligation), two maintained a residual tremor and one deteriorated, although tremors eventually resolved after 3 months. In one surgically treated cat that manifested tremors with PANS, neurological signs resolved within 3 weeks, but a residual pelvic limb tremor remained. Of the four cats treated only medically, three had their tremors resolved and one maintained a residual tremor.

## DISCUSSION

This is the first study to describe the clinical features of tremors in a population of cats diagnosed with HE‐CPSS or PANS. Tremor phenotype in cats with HE‐CPSS was variable, with approximately half of the population manifesting generalised tremors and the other half head tremors. Most tremors were episodic. Although intentionality was reported in one‐fifth of the population, most tremors occurred at rest.

Interestingly, tremors were the only neurological sign in one‐third of cats. Hepatic encephalopathy due to congenital portosystemic shunts should therefore be considered in the list of differential diagnoses in cats with tremors. Tremors resolved in most cats that received treatment for HE‐CPSS, and one cat with PANS that was medically treated. Not all cats had tremors at the time of admission; two cats manifested tremors after surgery as part of PANS semiology. Whether the cause of tremors in HE‐CPSS and PANS represent the same disease entity is unknown. The pathophysiology of tremors in HE‐CPSS and PANS in cats remains unknown. In dogs with HE‐CPSS that had surgical shunt attenuation, hepatic encephalopathy resolved but hypermanganesaemia persisted.^15^ Astrocytic lesions have been found in the cerebellum of dogs with HE‐CPSS,^16^ and therefore, a cerebellar origin of the tremors cannot be ruled out.

Asterixis or ‘flapping tremor’ is a hallmark sign in hepatic encephalopathy in people.^17^ Asterixis is an asynchronous, irregular and variable in frequency and amplitude negative myoclonus that usually affects the upper and/or lower limbs, and rarely the face, which is speculated to be associated with lesions in the thalamus and midbrain.^18^ Asterixis is non‐intentional, and it is a disorder of posture. To elicit the sign, the patient has to adopt a constant, specific posture against gravity, and therefore, it cannot be evaluated in cats.^18^

Holmes tremor (HT) is a rare symptomatic low frequency (<4.5 Hz) tremor present both at rest and with intention. A double lesion is required to develop HT, with both the dopaminergic nigrostriatal system and the cerebello‐thalamo‐cortical or dentate‐rubro‐olivary pathways being affected.^19^ All lesion locations appear to be connected to a common brain circuit including the red nucleus, thalamus, globus pallidus and cerebellum.^20^ Common aetiologies for HT include vascular encephalopathy and head trauma, but metabolic encephalopathy has not been reported.^19^

Parkinsonian‐like syndrome secondary to manganese accumulation in the basal nuclei can occur in manganese intoxication. In people, manganese preferentially accumulates in the globus pallidus, subthalamic nucleus, substantia nigra and striatum, but can also accumulate in the cerebellum, red nucleus, pons, cerebrocortex, thalamus and anterior horn of the spinal cord. This syndrome is characterised by limb hypertonicity, bradykinesia, rapid postural tremor and postural instability.^21^

Identifying the pathophysiology of tremors in cats with HE‐CPSS is challenging. Although asterixis could be present in cats with HE‐CPSS, we cannot instruct them to adopt specific postures, and therefore we are unable to assess for this in our patients. HT shares phenotypical similarities with tremors and pathways affected in one cat with HE‐CPSS from our study (thalamus, red, olivary, dentate nuclei). Parkinson's disease has not been recognised in animals^22^; however, parkinsonian‐like syndrome can be reproduced in rodents secondary to local or systemic administration of toxic drugs.^23^ Therefore, hypermanganesaemia in HE‐CPSS could potentially be related to a parkinsonian‐like syndrome in cats.

The limitations of this study include its retrospective nature, lack of video recordings and MRI of the brain in some cases and lack of electrophysiological tremor evaluation in all the cases.

In conclusion, tremor can be a neurological sign in cats with HE‐CPSS. However, no conclusions can yet be drawn regarding its pathophysiology. Future studies with video recordings should be pursued to improve our understanding of the phenomenology.

## AUTHOR CONTRIBUTIONS

*Conceptualisation, methodology, investigation, analysis and writing—original draft and review and editing*: Theofanis Liatis. *Methodology, analysis, review, editing and supervision*: Sofie F.M. Bhatti, Barbara Glanemann and Steven De Decker.

## CONFLICT OF INTEREST STATEMENT

The authors declare no potential conflicts of interest with respect to the research, authorship and/or publication of this article.

## FUNDING INFORMATION

The authors received no financial support for the research, authorship and/or publication of this article.

## ETHICS STATEMENT

No ethical approval was required for this study due to its retrospective nature.

## Supporting information



Supporting information

## Data Availability

The data that support the findings of this study are available from the corresponding author upon reasonable request.
