# Supplementary material for: Tremors in cats with hepatic encephalopathy‐congenital portosystemic shunts or postattenuation neurological syndrome
Source: Vet Rec. 2024 Nov 14;196(1):e4746. doi: 10.1002/vetr.4746 (PMC11698092; doi:10.1002/vetr.4746)
Supplement: Supplementary file 1 — Supporting information [file VETR-196-e4746-s001.docx]

**Supplementary material S1.** Signalment, presenting complaints, clinical and neurological findings, diagnosis and treatment in feline cases with hepatic encephalopathy due to congenital portosystemic shunts (HE-CPS) or post-attenuation neurologic syndrome (PANS) and tremors

| Case no | Breed | Sex | Age at presentation (y) | BW (kg) | Onset of signs | Progression | Lateralisation | Presenting complaints | Clinical findings | Neurological findings (localisation) | Tremors | Dx | Sx | Medical Tx | Tremor outcome at 1-month follow-up (type of follow-up) |
| --- | --- | --- | --- | --- | --- | --- | --- | --- | --- | --- | --- | --- | --- | --- | --- |
| *HE-CPSS* | | | | | | | | | | | | | | | |
| 1 | DSH | MN | 9.6 | - | C | 0 | 0 | Ataxia, mydriasis, hypersalivation, tremors | Low BCS | Cerebellar ataxia, nystagmus, mydriasis (cerebellar) | HT  Contin  Nonint | Single EH-CPSS | PL | Diet, lactulose, ampicillin, phenobarbital | NA as PTS after Sx due to deterioration |
| 2 | DSH | FN | 3 | - | C | 1 | 0 | Epileptic seizure, body weight loss, tremors | Low BCS, copper-coloured iris, | Tremors only (diffuse CNS) | GT  Episod  Nonint | Single EH-CPSS | PL | Diet, lactulose, phenobarbital | NA as LTF |
| 3 | BSH | MN | 1 | - | C | 1 | 0 | Epileptic seizure, ataxia, tremors | - | General proprioceptive vs bilateral vestibular ataxia, tetraparesis, PR deficits (multifocal) | HT  Episod  Nonint | Single EH-CPSS | PL | Diet, lactulose, ampicillin | NA as LTF |
| 4 | Persian | FN | 0.75 | - | C | 1 | 0 | Ataxia, hypersalivation, PU/PD, tremors | Microsomia | Vestibulocerebellar ataxia (vestibulocerebellar) | GT  Episod  Nonint | Single EH-CPSS | PL | Diet, lactulose, ampicillin, phenobarbital | Residual (re-exam) |
| 5 | DSH | ME | 0.5 | 1.93 | C | 1 | 0 | Anorexia, BW loss, lethargy, ataxia, tremors | Low BCS | Obtundation, vestibulocerebellar ataxia (vestibulocerebellar) | HT  Episod  Nonint | Single EH-CPSS | PL | Diet, lactulose, metronidazole | Resolved (re-exam) |
| 6 | Exotic short hair | MN | 7 | 3.2 | C | 1 | 0 | Anorexia, BW loss, lethargy, ataxia, hypersalivation, tremors | Low BCS, hypersalivation, poor skin/hair condition | Obtundation, vestibulocerebellar ataxia, ambulatory tetraparesis, PR deficits, equivocal menace response deficits (vestibulocerebellar) | GT  Episod  Nonint | Single EH-CPSS | ND | Diet, lactulose, amoxycillin-clavulate | Resolved (re-exam) |
| 7 | Maine Coone | ME | 0.5 | 2.8 | C | 1 | 0 | Anorexia, lethargy, tremors, hypersalivation, poor growth, poor coat, | Low BCS, copper-coloured iris, hypersalivation, poor skin/hair | Tremors only (diffuse CNS) | GT  Episod  Nonint | Single EH-CPSS | PL | Diet, lactulose, amoxycillin-clavulate | Resolved (re-exam) |
| 8 | DSH | FE | 0.5 | - | C | 1 | 0 | Anorexia, tremors, ataxia, hypersalivation, poor growth | Low BCS, copper-coloured iris, hypersalivation, microsomia | Obtundation, four-limb ataxia, menace reponse deficits (multifocal) | HT  Episod  Nonint | Single EH-CPSS | PL | Diet, lactulose, amoxycillin-clavulate | Resolved (re-exam) |
| 9 | DSH | FN | 0.58 | 2 | C | 1 | 1 | Ataxia, BW loss, tremors, hypersalivation, vomiting | Low BCS, hypersalivation, microsomia | Cerebellar ataxia, equivocal PR deficits, menace response deficits, anisocoria (cerebellar) | HT  Episod  Intent | Multiple EH-PSS | ND | Diet, lactulose, amoxycillin-clavulate | Residual (re-exam) |
| 10 | DSH | MN | 1.6 | 3.4 | C | 1 | 0 | Hypersalivation, tremors | Hypersalivation | Obtundation, vestibulocerebellar ataxia (vestibulocerebellar) | GT  Episod  Nonint | Single EH-CPSS | CL | Diet, lactulose, phenobarbital | Resolved (phonecall) |
| 11 | BSH | ME | 0.6 | 2.19 | C | 1 | 0 | Tremors, hypersalivation, poor growth | Hypersalivation, microsomia | Tremors only (diffuse CNS) | HT  Episod  Nonint | Single EH-CPSS | PL | Diet, lactulose, amoxycillin-clavulate, phenobarbital | Deteriorated at 1-month but resolved at 3 -months (phonecall) |
| 12 | DSH | FN | 0.5 | 1.53 | A | 1 | 0 | Tremors, ataxia, poor growth, abnormal behaviour | Microsomia | Obtundation, manic behaviour, four-limb ataxia, menace response deficits (multifocal) | GT  Episod  Nonint | Single IH-CPSS | PL | Diet, lactulose, amoxycillin-clavulate, levetiracetam | Resolved (re-exam) |
| 13 | DSH | MN | 1.1 | 3.25 | C | 1 | 0 | Lethargy, hypersalivation, tremors, poor growth | Low BCS, hypersalivation, copper-coloured iris, microsomia | Tremors only (diffuse CNS) | GT  Episod  Nonint | Single EH-CPSS | CL | Diet, lactulose, phenobarbital | NA as PTS after Sx due to deterioration |
| 14 | DSH | MN | 0.25 | 0.90 | C | 1 | 0 | Anorexia, lethargy, ataxia, tremors, hypersalivation | Low BCS, hypersalivation | Obtuntation, bilateral vestibular ataxia (vestibulocerebellar) | HT  Episod  Intent | Single EH-CPSS | PL | Diet, lactulose, amoxycillin-clavulate | Residual (re-exam) |
| 15 | DSH | FN | 3.5 | 3.5 | C | 1 | 0 | Tremors, seizures, lethargy, ataxia, hypersalivation | - | Obtuntation, vestibulocerebellar ataxia, ambulatory tetrapares, PR deficits (vestibulocerebellar) | HT  Episod  Nonintent with intentional features | Single EH-CPSS | ND | Diet, lactulose, levetiracetam | Resolved (re-exam) |
| 16 | BSH | ME | 0.33 | 2 | C | 1 | 0 | Tremors, hypersalivation | Low BCS, copper-coloured iris, hypersalivation | Tremors only (diffuse CNS) | TL  Episod  Nonint | Single EH-CPSS | ND | Diet, lactulose, amoxycillin-clavulate | Resolved (phonecall) |
| 17 | Bengal | MN | 0.5 | 3.4 | C | 1 | 0 | Tremors, lethargy, ataxia, tetraparesis | Microsomia | Obtuntation, general proprioceptive ataxia, ambulatory tetraparesis, menace response deficits OU, facial hypoaesthesia (multifocal) | GT  Episod  Nonint | Single EH-CPSS | PL | Diet, lactulose, phenobarbital, levetiracetam | Resolved (re-exam) |
| *PANS* | | | | | | | | | | | | | | | |
| 18 | DSH | FE | 0.5 | 1.16 | H | 0 | 0 | Tremors, blindness | - | Central blindness, menace response deficits, present PLR OU (multifocal) | GT  Contin  Nonint | Single EH-CPSS | PL | Diet, lactulose, phenobarbital, leveriracetam | Resolved (re-exam) |
| 19 | DSH | ME | 0.5 | 1.8 | H | 1 |  | Tremors, seizures | Microsomia | Tremors only (diffuse CNS) | GT  Episod  Nonint | Single EH-CPSS | CL | Diet, lactulose, amoxycillin-clavulate, phenobarbital, leveriracetam | NA as LTF |

M: male, F: female, N: neutered, y: year, m: month, C: chronic, A: acute, H: hyperacute, BW: body weight, BCS: body condition score, Sx: surgery, Dx: diagnosis, PL: partial ligation, CL: complete ligation, Tx: treatment, CNS: central nervous system, ND: not done, HT: head tremor, GT: generalised tremor, DSH: domestic short hair, BSH: British short hair, IH-CPSS: intrahepatic congenital portosystemic shunts, EH-CPSS: extrahepatic congenital portosystemic shunts; TL: thoracic limbs; NA: not available; LTF: lost to follow-up; PTS: put to sleep

**Supplementary material S2.** Diagnostic procedures performed in in feline cases with hepatic encephalopathy due to congenital portosystemic shunts (HE-CPS) or post-attenuation neurologic syndrome (PANS) and tremors

| Case no | CBC | Serum biochemistry | BAST | Ammonia | Abdominal ultrasound | Abdominal CT angiography | Intraoperative portovenography | Liver biopsy |
| --- | --- | --- | --- | --- | --- | --- | --- | --- |
| 1 | YES | YES | YES | YES | YES | NO | YES | YES |
| 2 | YES | YES | YES | YES | NO | NO | YES | YES |
| 3 | YES | YES | YES | NO | YES | NO | YES | YES |
| 4 | YES | YES | YES | YES | YES | NO | YES | YES |
| 5 | YES | YES | YES | YES | YES | NO | YES | YES |
| 6 | YES | YES | YES | YES | YES | NO | NO | NO |
| 7 | YES | YES | NO | YES | YES | NO | YES | NO |
| 8 | YES | YES | YES | YES | YES | NO | YES | YES |
| 9 | YES | YES | YES | YES | YES | NO | YES | YES |
| 10 | YES | YES | YES | NO | YES | NO | YES | YES |
| 11 | YES | YES | NO | YES | YES | NO | YES | NO |
| 12 | YES | YES | NO | YES | YES | YES | YES | YES |
| 13 | YES | YES | YES | YES | NO | YES | YES | NO |
| 14 | YES | YES | NO | YES | YES | YES | YES | NO |
| 15 | YES | YES | YES | YES | YES | NO | NO | NO |
| 16 | YES | YES | YES | NO | NO | YES | NO | NO |
| 17 | YES | YES | YES | YES | NO | YES | YES | YES |
| 18 | YES | YES | YES | YES | YES | NO | YES | YES |
| 19 | YES | YES | YES | YES | YES | NO | YES | YES |
